# Do mother-child conversations about safety differ in middle- and low-income families?

**DOI:** 10.5249/jivr.v11i2.1093

**Published:** 2019-07

**Authors:** Elizabeth E. O’Neal, Jodie M. Plumert

**Affiliations:** ^*a*^Department of Psychological and Brain Sciences, University of Iowa, Iowa City, IA, USA.

**Keywords:** Mother-child communication, Unintentional childhood injuries, Injury prevention, Low-income families

## Abstract

**Background:**

Children from low-income families experience a disproportionate number of unintentional injuries compared to their middle-income peers. Parents are well positioned to teach children about avoiding injury, yet little is known about parent-child safety conversations in low-income families. This study examined to what extent mother-child safety conversations differ between low- and middle-income families.

**Methods:**

Mothers and their 8- to 10-year-old children from low- and middle-income families discussed and rated the safety of photos showing another child engaged in potentially dangerous activities.

**Results:**

Dyads disagreed over safety ratings on a third of trials, and both middle- and low-income mothers were highly successful in resolving disagreements in their favor. Middle-income mothers justified their ratings by referring to almost twice as many dangerous features than outcomes, whereas low-income mothers generated roughly equal numbers of dangerous features and outcomes. Middle-income children did not differ in their references to dangerous features and outcomes, but low-income children focused heavily on dangerous outcomes relative to dangerous features.

**Conclusions:**

Describing how middle- and low-income families discuss safety is a first step in understanding whether similarities and differences contribute to how middle- and low-income children evaluate and navigate potentially dangerous situations.

## Introduction

Unintentional injury accounted for nearly 400 deaths and over 850,000 reported non-fatal injuries among 8- to 10-year-olds in the US in 2017.^1^ Past research has indicated that rates of injury tend to be higher for children from low-income families in comparison to middle-class families.^2,3^ Lower levels of education and increased exposure to injury risk have been identified as contributing to this trend. ^4,5^ As such, understanding what contributes to heightened injury risk in low-income populations is of primary concern to researchers, practitioners, and parents alike. Another factor that may play a role in this disparity is how parents talk to children about safety. Parents are a seemingly natural choice for teaching children about safety, due to the amount of time they spend with their children and the number of situations that parents and children encounter where injury is a possibility. Recent research has revealed that parents use conversations about safety as an opportunity to promote the internalization of safety values in their children.^6,7^ The current investigation sought to examine the extent to which these conversations differ between middle- and low-income families.

Much of the past work on protective factors for reducing injury risk has focused on parental supervision.^8,9^ However, constant parental supervision lessens after the second year^10^and continues to decline as children grow older,^8^ as does parents use of verbal directives.^10^ This decrease means that over time responsibility for regulating behavior shifts from parent to child, requiring children to control their own behavior when faced with potentially risky situations. One way parents can help children regulate their behavior is by teaching them safety rules (e.g., “never touch a hot stove”). There is some evidence to indicate that rules are associated with a marginal reduction in children’s risky behaviors.^11^ However, Morrongiello, Midgett, and Shileds^12^ reported that children aged 4 to 6 years spontaneously recalled only half of the home safety rules put in place by mothers and did not have a good understanding of the safety issues underlying these rules. Similarly, Peterson and Saldana^13^ found that even though parents regularly practiced home safety rule use with their 8-year-old children, these rules were not applicable in two-thirds of children’s reported injuries. Further, rules are typically implemented by parents as a reaction to a near or sustained injury, not as a means to proactively prevent injury.^14^

Another way in which parents can help children regulate their behavior is by teaching them how to independently evaluate potentially dangerous situations via shared social interactions during parent-child conversations about safety.^6,7^These conversations serve as a scaffold – a support for transferring knowledge and skills from older, more skilled individuals to younger, less knowledgeable individuals.^15^ Children play an active give-and-take role in these interactions, allowing parents to better evaluate their child’s current level of understanding and frame their message accordingly.^16^ This means first gaining an understanding of children’s knowledge about potentially risky activities and then tailoring conversations to address gaps in children’s safety knowledge, with the goal of helping children understand why particular situations might be dangerous. 

Recently, O’Neal, Plumert, and Peterson^7^ studied middle-income parent-child conversations about safety after a child had sustained a serious injury. When discussing how to prevent the injury from reoccurring in the future, parents were more likely to tell older children about why the activity was dangerous and were more likely to tell girls to be more careful in the future, indicating that parents use conversation to encourage future self-regulation. O’Neal and Plumert^6^ have also examined conversations about safety before a child has sustained an injury by asking middle-income mothers and their 8- and 10-year-old children to view and discuss photographs of another child engaged in potentially risky activities. Mothers promoted the internalization of safety values by pointing out dangerous features of an activity and the potentially dangerous outcomes that could result. Moreover, mothers capitalized on disagreements about an activity’s safety by increasing their references to dangerous features of the activity. This work indicates that highlighting causal connections between dangerous aspects of an activity and its possible unfavorable outcomes may play an important role in teaching children how to independently evaluate potentially dangerous situations. 

At present, almost nothing is known about how mothers and children from low-income households talk about safety. However, other research has shown that income differences in parent-child interactions impact reading ability and school readiness.^17,18^ For example, Portes^18^ found that while maternal verbal guidance predicted better mathematics, language, and reading abilities in 5th and 6th grade children, this kind of verbal guidance was observed much less often among the low-income mothers enrolled in the study. Reading also benefits children’s school readiness^19^ and recent interventions aimed at improving parent-child interactions while reading have shown positive results among low-income families. ^17^ As a first step in understanding the possible links between parent-child conversations about safety and injury risk in low-income populations, we sought to determine whether mother-child conversations about safety differ between middle-income and low-income families. Specifically, we were interested in how the two groups might differ in 1) the perceived safety of the activities, 2) the input that mothers requested from their children, and 3) the rationales that mothers and children used to justify their safety ratings when they agreed and disagreed about the safety of the activity. We also examined whether there were income differences in children’s injury history.

## Methods 

**Participants**

One hundred and six mothers and children from low-income (N = 20 dyads) and middle-income (N = 33 dyads) families participated in the study. Families were recruited for the two groups based on whether they qualified for government assistance (see Table 1 for demographic information). Significant differences emerged between the two groups on reported education level, t (32) = 4.99, p <.001, and income, t (23) = 8.35, p <.001 (note that not all mothers reported income). Children were recruited from a child research participant registry maintained by the Department of Psychological and Brain Sciences at the University of Iowa and from flyers posted throughout the community with an emphasis on outlets that serve low-income populations. Consent and assent was obtained from mothers and children, respectively. The institutional review board at the University of Iowa approved the study. Children received a $10 gift card and mothers received a $25 gift card to a local retailer for their participation. 

**Table 1:** Demographic information for low- and middle-income groups.

	Income Group	
	Low-Income(N = 20 dyads)	Middle-Income (N = 33 dyads)
**Child age (years)**	8.9 (.84)	8.9 (.85)
**Child gender (% male)**	55	35
**Child ethnicity (%)**		
Caucasian	50	93
African-American	25	0
Hispanic	10	0
American Indian	5	0
Multirace	10	6
**Maternal education (%)**		
Doctoral degree	0	3
Master degree	0	36
4-year degree	10	30
2-year degree	20	6
Some college	40	18
High school diploma	25	6
Eighth grade	5	0
**Household income (%)**		
Less than $14,999	25	0
$15,000 – $29,999	10	0
$30,000 - $59,999	25	1
$60,000 - $89,999	0	24
$90,000 – $119,999	0	21
More than $120,000	0	33
Not reported	40	9

**Apparatus and Materials**

Participants sat in front of a 46-inch (116.8 cm) touch screen monitor at a comfortable viewing distance. Two camcorders videorecorded the mother-child conversations from side and back angles. We used two sets of 14 photographs from O’Neal and Plumert.^6^ Each depicted a similarly aged, gender-matched Caucasian child engaged in various physical activities. Two photographs were used for familiarization and 12 photographs were used for testing. 

**Design and Procedure**

**Figure 1:**
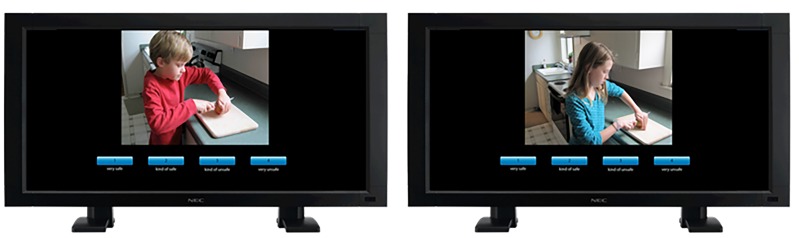
Examples of the gender-matched photos and Likert scale used in the rating tasks.

The session took approximately 45 minutes and included two phases. In the first phase, mothers and children independently viewed and rated the randomly ordered set of photographs in separate rooms. Each was first familiarized with how to use the touchscreen using two additional photos of safe and unsafe activities. For each photograph, mothers and children rated the safety of the activity on a 4-point Likert scale: 1) very safe, 2) kind of safe, 3) kind of unsafe, and 4) very unsafe (Figure 1). In the second phase, mothers and children viewed each photograph again and were asked to discuss and come to a decision together about the safety rating of each activity. Parents also reported any unintentional injuries sustained by the child that required professional medical attention using the Accidental Injury Questionnaire (AIQ).^20^ This questionnaire lists 12 types of injury (e.g., broken bones, cuts, sprains) and asks parents to indicate how many times their child had previously sustained each type of injury. 

**Coding and Measures**

Mother-child conversations were transcribed verbatim. Coding of the transcripts detailed how the joint safety rating decision was made and how mothers and children justified their safety ratings. 

We coded whether the mother prompted the child to provide a safety rating and whether the child provided the first rating. Additionally, we coded whether disagreement over the rating arose during the conversation and whether the disagreement was resolved in favor of the mother. Scores for each of these measures represented the percentage of occurrences across the 12 photographs. 

We separately coded the rationales that mothers and children used to support their ratings. Most rationales referred to dangerous features (directly observable aspects of the photograph; “The handle of that pot is probably hot.”) and outcomes (possible dangerous consequences of the activity; “She could burn her hand.”) of the activity. Dyads also made references to other rationales which were not analyzed further due to their limited occurrence. A majority of rationales conveyed new (88%) rather than repeated information (12%) about the activity. Mother and child rationale scores represented the mean number of dangerous feature and outcome references per photograph. We also coded whether the child provided the first rationale and whether mothers prompted children to provide a rationale to support their rating. Scores for these two measures represented the percentage of occurrences across the 12 photographs. 

Individual safety rating scores (averaged over the 12 photographs) were calculated separately for mothers and children, and joint safety rating scores (averaged over the 12 photographs) were calculated for dyads.

Scores on the AIQ represented the total number of reported unintentional injuries that required professional medical attention.

**Interrater Reliability**

Interrater reliabilities between coders were calculated on 15 dyads (28% of the sample). Reliabilities between coders for the number of references mothers and children each made to dangerous features and outcomes were calculated using intraclass correlations (ICC), and ranged between ICC = .78 and .95. Reliabilities for the measures of whether mothers prompted children for ratings and rationales, whether children provided the initial rating and rationale, whether there was disagreement over the rating, and whether disagreements were resolved in the mother’s favor were calculated using Cohen’s kappa and ranged between K = .65 and 1.00, with kappas on 5 of the 6 measures at .80 or larger. Coders were unaware of dyads’ income status. 

## Results

**Past Injury History **

A Mann-Whitney test revealed that mothers reported significantly more injuries requiring professional medical attention for low-income (Mdn = 1.00, range = 0 – 4) than middle-income children (Mdn = 0.00, range = 0 – 6), U = 199.00, p = .01. 

**Safety Ratings**

***Individual Safety Ratings. ***While all mothers and children rated the activities to be “kind of unsafe” on average, analysis of the safety ratings mothers and children made independently prior to the joint rating task revealed that low-income children rated the activities as more unsafe (M = 3.36, SD = .45) than did the middle-income children (M = 3.10, SD = .35), t (51) = -2.34, p = .02. Likewise, low-income mothers rated the activities as more unsafe (M = 3.47, SD = .36) than did the middle-income mothers (M = 3.27, SD = .32), t (51) = -2.12, p = .04.

***Joint Safety Ratings. ***Middle-income children (M = 87% of trials, SD = 15) were more likely than low-income children (M = 77% of trials, SD = 22) of trials to provide the first rating, t (51) = -2.01, p = .05. There was no difference in the percentage of trials that middle-income mothers (M = 33%, SD = 25) and low-income mothers (M = 33%, SD = 25) prompted children to provide the first rating, t (51) = -.57, ns. 

***Disagreements about ratings. ***Once one dyad member offered a safety rating, the dyad typically started to discuss the safety of the activity. Middle income mother-child dyads disagreed on safety ratings on 32% of trials (SD = 14), and low-income mother-child dyads disagreed on 28% of trials (SD = 19), t (51) = .90, ns. When there was disagreement, middle-income and low-income mothers gave more conservative ratings than their children on 62% (SD = 33) and 66% (SD = 32) of trials, respectively, t (51) = -.44, ns. Mothers from both groups resolved disagreements in their favor on a majority of trials, with middle-income mothers doing so on 71% (SD = 27) of trials and low-income mothers doing so on 78% (SD = 34) of trials, t (51) = -.94, ns. 

***Final joint safety ratings. ***Comparison of the mean final safety ratings revealed that low-income dyads (M = 3.50, SD = .34) again rated the activities as more unsafe than did middle-income dyads (M = 3.29, SD = .25), t (51) = -2.34, p = .02. Given that disagreements were typically resolved in favor of the mother, we also wanted to know how closely the final joint rating aligned with mothers’ earlier individual rating. The correlation between mothers’ individual ratings and the final joint safety rating was significant both for low-income dyads, r = .74, p <.001, and for the middle-income dyads, r = .63, p <.001. 

**How Did Mothers and Children Justify Their Ratings?**

A central element of the mother-child discussions were the rationales mothers and children provided to justify their safety ratings. 

***First Rationale. ***Children from middle income families provided the first rationale on 59% (SD = 24) of trials, and children from low-income families did so on 49% (SD = 28) of trials, t (51) = -1.46, ns. 

***Maternal Prompting. ***Mothers sometimes prompted children to justify their rating as a means of gaining more information about their child’s evaluation of the activity (“tell me why you think that”). We entered the proportion of agreement and disagreement trials on which mothers prompted their children into an Income Status (middle-income, low-income) x Trial Type (agreement, disagreement) mixed model ANOVA. This analysis revealed no significant difference between middle- (M = .46; SD = .31) and low-income (M = .44; SD = .43) mothers, F (1, 50) = .04, ns, and no effect of trial type, F (1, 50) = 1.47, ns. 

***Safety Rating Rationales. ***To determine whether the references that mothers and children used to support their ratings varied by income or disagreement we conducted separate Income Status (middle-income, low-income) x Rationale Type (feature, outcome) x Trial Type (agreement, disagreement) mixed model ANOVAs for references to dangerous features and outcomes. We conducted separate analyses for mothers and children to examine their individual contributions to safety conversations. 

**Figure 2:**
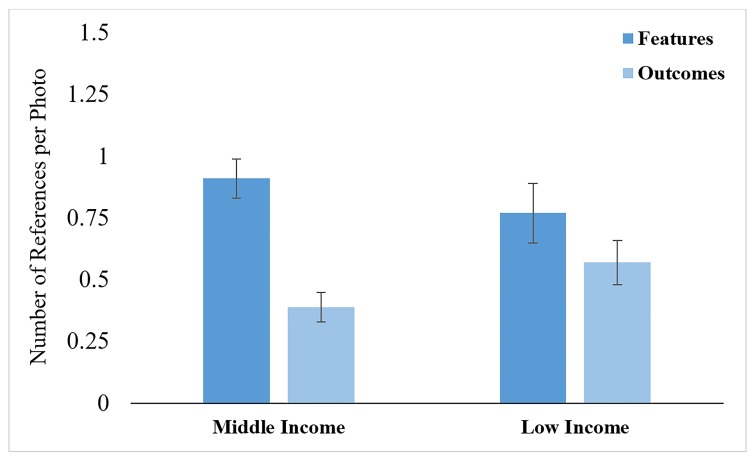
Mean number of middle- and low-income mothers’ references to dangerous features and outcomes.

***Mothers. *** There was a significant effect of trial type, F (1, 50) = 31.30, p <.001, ηp^2= .39. Mothers provided more rationales overall to support their rating when there was disagreement (M = 1.00 SD = .86) than agreement (M = .48, SD = .40). There was also an effect of rationale type, F (1, 50) = 16.40, p <.01, ηp^2= .25, which was subsumed under a significant Income Status x Rationale Type interaction, F (1, 50) = 4.34, p = .04, ηp^2= .08. Simple effects tests revealed an effect of rationale type for middle-income mothers, F (1, 31) = 20.05, <.001, η^2 = .40, but not for low-income mothers, F (1, 19) = 1.18, ns. Middle-income mothers made significantly more references to dangerous features than to dangerous outcomes (Figure 2). However, low-income mothers did not differ in this regard.

**Figure 3:**
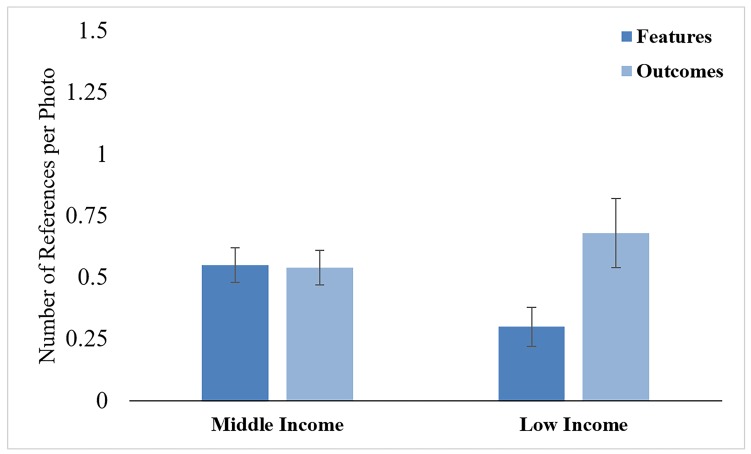
Mean number of middle- and low-income children’s references to dangerous features and outcomes.

***Children. ***There was an effect of rationale type, F (1, 50) = 7.25, p = .01, ηp^2= .13, which was subsumed under a significant Income Status x Rationale Type interaction, F (1, 50) = 6.81, p = .01,ηp^2= .31. There was an effect of rationale type for low-income children, F (1, 19) = 6.77, p = .02, η^2= .06, but not for middle-income children, F (1, 31) = .83, ns. Low-income children referred to significantly more dangerous outcomes than dangerous features, whereas middle-income children did not differ in this regard ( Figure 3).

## Discussion

We investigated potential income differences in mother-child conversations about safety by asking mothers and children from low- and middle-income families to discuss and rate a set of photographs which depicted another child engaged in various physical activities. There were many similarities across low-income and middle-income dyads. Mothers from both groups were equally likely to prompt children to provide the initial rating and to justify their initial rating. Similarly, mothers and children from low- and middle-income families disagreed over their initial safety ratings on roughly a third of all trials and both low- and middle-income mothers successfully resolved disagreements in their own favor. In addition, the final joint safety ratings were highly correlated with mothers’ individual safety ratings in both middle- and low-income groups. These findings closely replicated those reported in the O’Neal and Plumert^6^ study with middle-income mother-child dyads.

Despite these similarities, the two groups differed in several key ways. Middle-income children were more likely to provide the initial safety rating than lower-income children. This is likely provided mothers of middle-income children with important information about gaps in their child’s current level of understanding about safety, a key ingredient for gearing scaffolding (i.e., support for the transfer of skills from an older, more experienced individual to a younger, less skilled individual) to the developmental level of the child.^15,16^ By providing the initial safety rating more often themselves, low-income mothers may have inadvertently missed opportunities for assessing their child’s level of understanding about safety. This, in turn, could contribute to less well-developed skills in evaluating potentially dangerous situations in lower-income populations. However, more work is needed to determine how these differences might impact injury risk in this population. 

How dyads perceived the safety of the activities also differed by income status. Interestingly, mothers and children from low-income families independently and jointly rated the activities as more unsafe than did middle-income mothers and children. Mothers and children from lower-income families may have perceived the activities as more unsafe because the lower-income children had actually experienced more injuries requiring medical attention than had the middle-income children. Possibly, the lower-income children had engaged in some of the types of potentially dangerous activities depicted in the photos and were more familiar with the adverse outcomes that could result.

The pattern of references to dangerous features and outcomes also differed between the middle- and low-income mothers and children. Middle-income mothers referenced more than double the number of dangerous features than outcomes, whereas there was no significant difference between low-income mothers’ references to dangerous features and outcomes. Middle-income children referred to dangerous features and outcomes roughly equally, while low-income children referred much more often to dangerous outcomes. This pattern suggests that low-income dyads focused more on the activities’ potential adverse outcomes than on the activities’ dangerous features that might lead to those adverse outcomes. 

Why might this be the case? Increased attention to potential adverse outcomes could again be tied to the fact that children from low-income families had experienced a greater number of medically-attended injuries. While important, focusing on dangerous outcomes without pointing out the numerous features that could lead to such outcomes could be another missed opportunity to help children internalize safety values. Research on how middle-income parents teach children about science phenomena has shown that parents often make causal connections between actions and their outcomes.^21^ In the case of teaching children about safety, making causal connections could help children better understand the connection between dangerous features and adverse outcomes.^6^

There are several limitations to the current study. First, there was limited diversity in our middle-income group compared to the low-income group, which may have influenced some of the reported group differences. Second, we do not know whether the safety ratings reflect how safe or unsafe mothers and children would perceive these activities to be outside of the lab, as demand characteristics likely influenced dyads to rate the activities as being more unsafe. Third, we do not know whether reported conversational differences are causally related to increased injury risk in low-income populations. 

Parents are ideally suited to teach children about safety, yet their contribution to safety via conversation is not well understood. The current study represents a first step in describing similarities and differences between how middle- and low-income mothers and children talk about safety. An important second step is to test whether these similarities and differences in safety conversations influence how low- and middle-income children evaluate and navigate potentially dangerous situations. 
